# Pin1 is required for sustained B cell proliferation upon oncogenic activation of Myc

**DOI:** 10.18632/oncotarget.7846

**Published:** 2016-03-02

**Authors:** Luana D'Artista, Andrea Bisso, Andrea Piontini, Mirko Doni, Alessandro Verrecchia, Theresia R. Kress, Marco J. Morelli, Giannino Del Sal, Bruno Amati, Stefano Campaner

**Affiliations:** ^1^ Center for Genomic Science of IIT@SEMM, Fondazione Istituto Italiano di Tecnologia (IIT), Milan, Italy; ^2^ Department of Experimental Oncology, European Institute of Oncology (IEO), Milan, Italy; ^3^ Laboratorio Nazionale CIB (LNCIB), Area Science Park, Trieste, Italy; ^4^ Dipartimento di Scienze della Vita, Università degli Studi di Trieste, Trieste, Italy

**Keywords:** c-myc, Pin1, lymphoma, proliferation

## Abstract

The c-*myc* proto-oncogene is activated by translocation in Burkitt's lymphoma and substitutions in codon 58 stabilize the Myc protein or augment its oncogenic potential. In wild-type Myc, phosphorylation of Ser 62 and Thr 58 provides a landing pad for the peptidyl prolyl-isomerase Pin1, which in turn promotes Ser 62 dephosphorylation and Myc degradation. However, the role of Pin1 in Myc-induced lymphomagenesis remains unknown. We show here that genetic ablation of *Pin1* reduces lymphomagenesis in Eμ-*myc* transgenic mice. In both *Pin1*-deficient B-cells and MEFs, the proliferative response to oncogenic Myc was selectively impaired, with no alterations in Myc-induced apoptosis or mitogen-induced cell cycle entry. This proliferative defect wasn't attributable to alterations in either Ser 62 phosphorylation or Myc-regulated transcription, but instead relied on the activity of the ARF-p53 pathway. Pin1 silencing in lymphomas retarded disease progression in mice, making Pin1 an attractive therapeutic target in Myc-driven tumors.

## INTRODUCTION

Pin1 is a Peptidyl-prolyl isomerase (PPIase) of the parvulin-like family that catalyzes the cis/trans isomerization of Prolines in peptide chains [1]. Among PPIases, Pin1 shows unique specificity, in that it specifically recognizes Prolines followed by a phosphorylated Ser or Thr residue [2, 3]. Proline-directed phosphorylation sites are frequent in the cellular proteome and are the targets of several kinases families, including Cyclin-dependent kinases, MAP-kinases, Polo-like kinases, Glycogen Synthase Kinase-3 and p38 kinases. Consistent with such high level of complexity, Pin1 has profound impact on a variety of fundamental biological processes [4].

A well-characterized Pin1 substrate is the Myc transcription factor. Pin1 is involved in an intricate phosphorylation/de-phosphorylation cycle that regulates Myc turnover. This regulatory cycle is ignited by ERK mediated phosphorylation of Ser 62, which enables GSK-3b dependent phosphorylation of Thr 58 [5, 6]. The double phosphorylated form of Myc is bound by Pin1, which allows subsequent de-phosphorylation of Ser 62 and ubiquitin dependent proteasomal degradation of Myc [7]. Consistent with this model, mutations in Thr 58 stabilize Myc [5, 8].

Myc is an oncogenic factor that is generally over-expressed in tumors: this can happen either indirectly through the activation of oncogenic pathways that stabilize Myc and/or augment c-*myc* transcription, or directly through amplification or translocation of c-*myc* gene, such as in Burkitt' s lymphoma (BL). Most remarkably, in BL, the open-reading frame of the translocated c-*myc* allele is frequently the target of secondary missense mutations [9]. Adoptive gene transfer experiments in the mouse hematopoietic system directly demonstrated that mutations at codon 58 (one of the hotspots in BL) augment the oncogenic potential of c-*myc* [10]. On this basis, one might hypothesize that, like Thr 58 mutation, a decrease in Pin1 activity should potentiate the oncogenic action of Myc: this putative tumor suppressive effect of Pin1 might be further reinforced by its positive action on p53 [11, 15], a key suppressor of Myc-induced lymphomagenesis [16].

The above notwithstanding, other effects of Pin1 would lead one to predict a positive role for this enzyme in Myc-induced lymphomagenesis. In particular, the direct action of Pin1 on Myc may positively modulate its transcriptional activity, either by favoring its interaction with co-activators such as p300 [17], or by augmenting its dynamic turnover on target genes [18]. Pin1 may also indirectly favor Myc activity, for example by promoting the degradation of Fbw7 [19], a ubiquitin ligase that contributes to Myc turnover [20, 21].

Using mouse genetics, we show that Pin1 is critical for efficient Myc-induced lymphomagenesis. This, however, cannot be accounted for by any of the aforementioned mechanisms. Instead, we report that Pin1 is required to avert the onset of an Arf-p53 dependent cytostatic response following Myc activation. Finally, based on a reverse-genetics approach, we provide proof-of-principle experiments validating Pin1 as a therapeutic target in Myc-driven lymphoma.

## RESULTS

**Figure 1:**
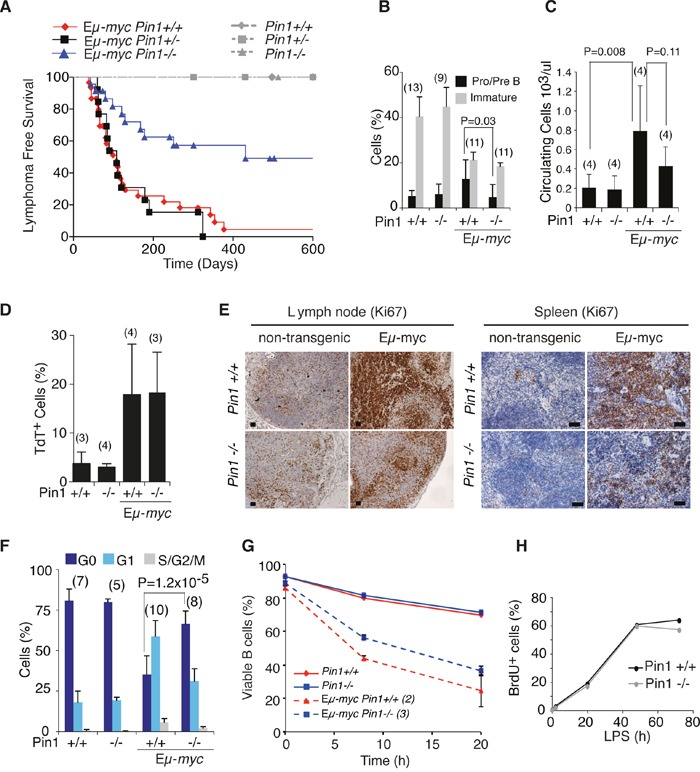
Lymphomagenesis and pre-tumoral analysis of Eμ-*myc Pin1−/−* mice **A.** Lymphoma-free survival in cohorts of Eμ-*myc* or control mice of the indicated Pin1 genotypes. The Median survival was 108 days for Eμ-*myc Pin1^+/+^* (N=30), 431 days for Eμ-*myc Pin1^−/−^* (N=23), and 108 days for Eμ-*myc Pin1^+/−^* (N=13). P-values were calculated with the log-rank (Mantel-Cox) test: P = 0.001 for Eμ-*myc Pin1^−/−^* vs. Eμ-*myc Pin1^+/+^*; P = 0.002 for Eμ-*myc Pin1* ^−/−^ vs. Eμ-*myc Pin1^+/−^*; P = 0.7287 for Eμ-*myc Pin1^+/+^* vs. Eμ-*myc Pin1^+/−^*. No lymphomas arose in non-transgenic mice, regardless of their *Pin1* genotype. In B-F, six weeks old Eμ-*myc* pre-tumoral mice and age matched non-transgenic mice were analyzed. **B.** Flow cytometric analysis of circulating B cell populations. Pro/Pre B lymphocytes are defined as B220^+^IgM^−^, Immature B lymphocytes as B220^+^IgM^+^ cells. **C.** Numbers of circulating lymphocytes in the peripheral blood of mice of the indicated genotypes, determined with a Hematological analyzer (Beckman Coulter). **D.** Percentage of apoptotic cells among splenic B220^+^ lymphocytes of the indicated genotypes, as assessed by Tunel assay. **E.** Sections of the indicated genotypes were stained with the proliferation marker Ki67. 3-4 mice of each genotype were analyzed. Representative sections are shown. Scale bars: 100 μm. **F.** Cell cycle distribution of circulating B cells, analyzed as described in Supplementary Figure 1E. **G.** Viability of splenic B cells purified from healthy Eμ*-myc* or non-transgenic mice, cultured in the absence of cytokines. After 8 and 20 hours, cells were stained with Propidium Iodide (PI) to exclude dead cells. The percentage of viable PI negative cells, measured by flow cytometry is reported. **H.** Cell cycle entry and proliferation of purified B cells cultured *in vitro* in the presence of LPS, as assessed by continuous labeling with BrdU. In B-D, F, average values and standard deviations are reported, based on the numbers of samples indicated in above the bars. P-values were calculated using Student's t-test.

To address the role of Pin1 in Myc-induced lymphomagenesis, we bred *Pin1* knockout mice [22, 23] with Eμ-*myc* transgenic mice [24]. Eμ-*myc Pin1^+/+^* and Eμ-*myc Pin1^+/−^* mice developed lymphomas with similar latency (average onset: 108 days) and penetrance (86% and 92% respectively). Eμ-*myc Pin1^−/−^* mice, instead, showed enhanced latency (431 days) and reduced penetrance (52%) (Figure 1A). This did not merely follow from a primary defect in B cell development, as *Pin1^−/−^* mice showed normal formation of bone marrow common myeloid/lymphoid progenitors (B220^−^IgM^−^CD25^−^c-kit^+^) and differentiation to Pro B (B220^+^IgM^−^CD25^−^c-kit^+^), Pre B (B220^+^IgM^−^CD25^+^c-kit^−^) (Supplementary Figure 1A) and immature B cells (B220^+^IgM^+^) (Figure 1B and Supplementary Figure 1B-1D). Hence, loss of Pin1 limits Myc-induced lymphomagenesis.

At the pre-tumoral stage, Eμ-*myc* mice display a characteristic increase in circulating Pro/Pre B cells and a concomitant reduction in immature B cells (Figure 1B and Supplementary Figure 1B) [24, 25]: while this differentiation block was still present in young Eμ-*myc Pin1^−/−^* mice, these animals showed significantly lower accumulation of Pro/Pre B cells (Figure 1B and Supplementary Figure 1B) and, as a consequence, decreased expansion of total circulating B cells (Figure 1C). Reduced expansion of the Pro/Pre B cell compartment was also observed in the bone marrow and in the spleen of Eμ-*myc Pin1^−/−^* mice (Supplementary Figure 1C, 1D). Another feature of the pre-tumoral stage in Eμ-*myc* mice is the co-occurrence of Myc-induced apoptosis and proliferation [26, 27]: these effects were dissociated in Eμ-*myc Pin1^−/−^* animals, which displayed normal induction of apoptosis (Figure 1D), but a defective proliferative response (Figure 1E, 1F and Supplementary Figure 1E). Culturing of control and pre-tumoral Eμ-*myc* B cells *ex vivo* confirmed that the apoptotic activity of Myc was preserved in the *Pin1^−/−^* background (Figure 1G). In contrast with the proliferative defect of Eμ-*myc Pin1^−/−^* B cells *in vivo*, primary lymphocytes isolated from non-transgenic *Pin1^−/−^* or *Pin1^+/+^* mice entered the cell cycle and proliferated with comparable efficiencies following stimulation with LPS *ex vivo* (Figure 1H and Supplementary Figure 1F), showing that *Pin1^−/−^* B cells have no intrinsic proliferative defect.

**Figure 2:**
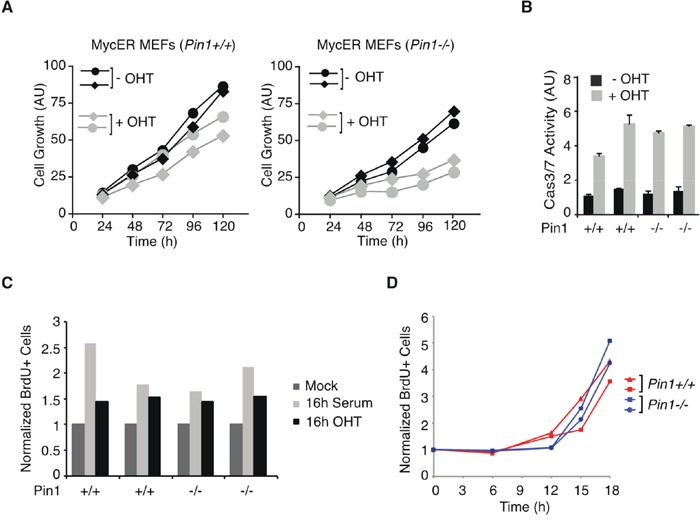
Pin1 is required for Myc driven proliferation in MEFs **A.** Growth of MycER-expressing MEFs [54] cultured in the presence or absence of OHT. Each curve represent independent low passage MEFs isolates of the indicated genotypes. (AU) arbitrary units. Although population doublings were already reduced upon OHT treatment in wild-type MEFs due to Myc-induced apoptosis, sustained MycER activation markedly reduced the expansion of *Pin1^−/−^* cultures [27, 30]. **B.** Apoptosis in MycER-expressing MEFs assessed by Caspase 3/7 activity. In A., B. data represent the average of a triplicate measure expressed as arbitrary units (AU). **C.** Serum-starved MycER-expressing MEFs cells were stimulated with either 20% serum or OHT, as indicated. After 16 hours MEFs were pulse-labeled with BrdU for 20 min. The percentage of BrdU-positive cells was determined by FACS. **D.** Cell cycle entry and proliferation of MEFs upon serum release, as assessed by continuous labeling with BrdU. In C., D. values represent the average from two independent samples.

The effect of Pin1 deletion observed *in vivo* in B cells was corroborated in mouse embryo fibroblasts (MEFs) expressing a 4-hydroxy-tamoxifen (OHT)-inducible MycER chimera: here, continuous MycER activation in proliferating cells preferentially suppressed the growth of *Pin1^−/−^* relative to *Pin1^+/+^* populations, while inducing apoptosis to similar extents with either genotype (Figure 2A, 2B). Of note, *Pin1^−/−^* MEFs proliferated less than wild-type, reflecting the pleiotropic role of Pin1 in regulating cell cycle progression [2, 4]. When serum-starved and subsequently induced to enter the cell cycle by treatment with either serum or OHT, *Pin1^−/−^* and *Pin1^+/+^* MEFs entered S-phase with similar efficiencies and kinetics (Figure 2C, 2D), showing that Pin1 was dispensable for re-entry into the cell cycle under the control of either endogenous or exogenous Myc [28, 29], and hence for the mitogenic activity of Myc *per se*. Altogether, the above data indicate a specific requirement of Pin1 for sustained proliferation following oncogenic activation of Myc in either B cells or fibroblasts.

The proliferative defect of *Pin1^−/−^* cells was reminiscent of previous observations on Cdk2, deletion of which caused Myc-overexpressing cells to undergo cellular senescence [30]. Since Cdk2 phosphorylation sites are also Pin1 consensus sites [31], we hypothesized that Cdk2- and Pin1-nullizygosity may cause similar defects in response to oncogenic Myc. However, unlike what was observed in Eμ-*myc Cdk2^−/−^* B cells [30], the mRNAs encoding the Cdk inhibitors p16^INK4a^ and p21^Cip1^ were not significantly up-regulated in the *Pin1^−/−^* background (Supplementary Figure 2A) and these proteins remained below detection in all samples (data not shown). As expected [16], the p19^ARF^ mRNA and protein accumulated in Eμ-*myc Pin1^+/+^* B cells accompanied by increased p53 levels, effects that were reduced in the *Pin1^−/−^* background (Supplementary Figure 2A, 2B). Consistent with these results, the quiescent state of Eμ-*myc Pin1^−/−^* B cells was not associated with enhanced expression of a panel of p53 target genes (Supplementary Figure 2C). Unlike in Eμ-*myc Cdk2*^−/−^ B cells [30], we were unable to detect accumulation of Senescence-associated Δ-Galactosidase (SA-Δ-gal) activity in Eμ-*myc Pin1^−/−^* cells (data not shown). Reactive oxygen species (ROS), the DNA damage response (DDR) marker γH2AX and phosphorylation of p53 on Ser 15 are stress-associated features that have also been linked with cellular senescence [32]. Consistent with previous reports [33–35] all of these features were elevated in pre-tumoral B cells relative to non-transgenic controls, but were not affected by the Pin1 genotype (Supplementary Figure 2D, 2E). Finally, senescence may also follow from telomere dysfunction [36], and loss of Pin1 has been associated with telomere shortening and premature aging in mice [37]: however, no significant telomere shortening occurred in mouse B cells upon either Myc expression, Pin1 loss, or both together (Supplementary Figure 2F). Altogether, our data lend no support for a mechanistic link between senescence and the proliferative defect of Eμ*-myc Pin1^−/−^* B cells.

**Figure 3:**
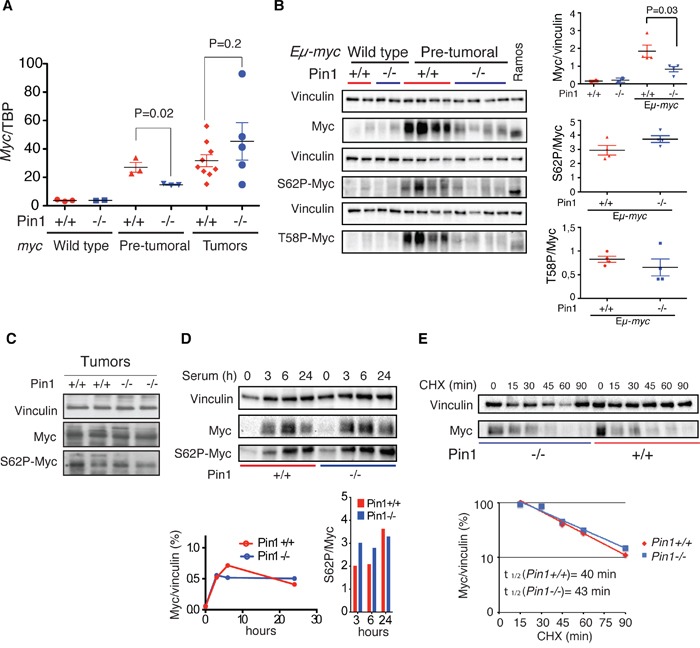
Loss of Pin1 does not affect Myc protein stability in B cells and MEFs **A.** c-*myc* mRNA levels normalized to the TBP mRNA (TATA Box Binding Protein) in control, pre-tumoral splenic B cells and Eμ-*myc* tumors of the reported Pin1 genotype, as assessed by RT-qPCR. **B.** Immunoblot analysis of total (Myc), Ser 62-phosphorylated Myc (S62P-Myc) and Thr 58-phosphorylated Myc (T58P-Myc) in B220+ cells purified from spleens of the indicated genotype. Vinculin is shown as loading control. Relative densitometric analysis was performed using the Image Lab 5.0 software. The levels of total Myc normalized to Vinculin, and of S62P-Myc and T58P-Myc normalized to total Myc are reported on the right. In A., B. P-values were calculated using Student's t-test. **C.** Immunoblot analysis of total Myc and S62P-Myc in Eμ-*myc* tumors of the reported Pin1 genotype. **D.** Immunoblot analysis of endogenous Myc in quiescent MEFs (0h) or the same cells stimulated with 20% serum for the indicated times. Relative quantifications, performed as described in B, are reported at the bottom, for either total Myc (left) or S62P-Myc (right). **E.** Stability of endogenous Myc in asynchronous proliferating *Pin1^+/+^* and *Pin1^−/−^* MEFs. Lysates were prepared at the indicated times after treatment of the cells with cycloheximide (CHX) and immunoblotted for total Myc and vinculin. The quantifications of the immunoblot and calculated Myc half-lives are shown at the bottom. Two independent MEF populations of each genotype were analyzed. One representative experiment is shown.

Survey of Myc mRNA and protein levels in pre-tumoral Eμ-*myc* B cells revealed a substantially lower accumulation in the *Pin1^−/−^* mutant background (Figure 3A, 3B). Yet, Myc was clearly active in Eμ-*myc Pin1^−/−^* cells, as apoptosis and blockade of B cell differentiation were induced as efficiently as in control Eμ-*myc Pin1^+/+^* cells (Figure 1B, 1D, 1G): given that induction of apoptosis requires higher Myc levels than the proliferative response [38], the phenotype of Eμ-*myc Pin1^−/−^* B cells was unlikely to follow merely from an overall decrease in Myc activity. At odds with pre-tumoral samples, the absence of Pin1 did not affect c-*myc* mRNA and Myc protein levels in Eμ-*myc* tumors (Figure 3A, 3C). We conclude that decreased c-*myc* expression most likely followed from the proliferative arrest of pre-tumoral B cells, which tumors had bypassed.

Since Pin1 can favor Ser 62 dephosphorylation and Myc degradation [5, 7, 39], its loss would be expected to lead to Myc accumulation, with a relative enrichment of the phospho-Ser 62 (S62P) form. However, the ratios of S62P to total Myc were independent of the Pin1 genotype in either Eμ-*myc* B cells or MEFs (Figure 3B-3D). Accordingly, T58P to total Myc also showed no significant alteration (Figure 3B). Total and S62P Myc also accumulated with comparable kinetics following mitogenic stimulation in *Pin1^+/+^* and *Pin1^−/−^* MEFs (Figure 3D), and the protein showed similar decay rates upon translational blockade with cycloheximide (CHX) (Figure 3E). Altogether, the phenotypes of *Pin1^−/−^* cells reported here were not directly linked to a deregulation of Myc protein phosphorylation and stability.

**Figure 4:**
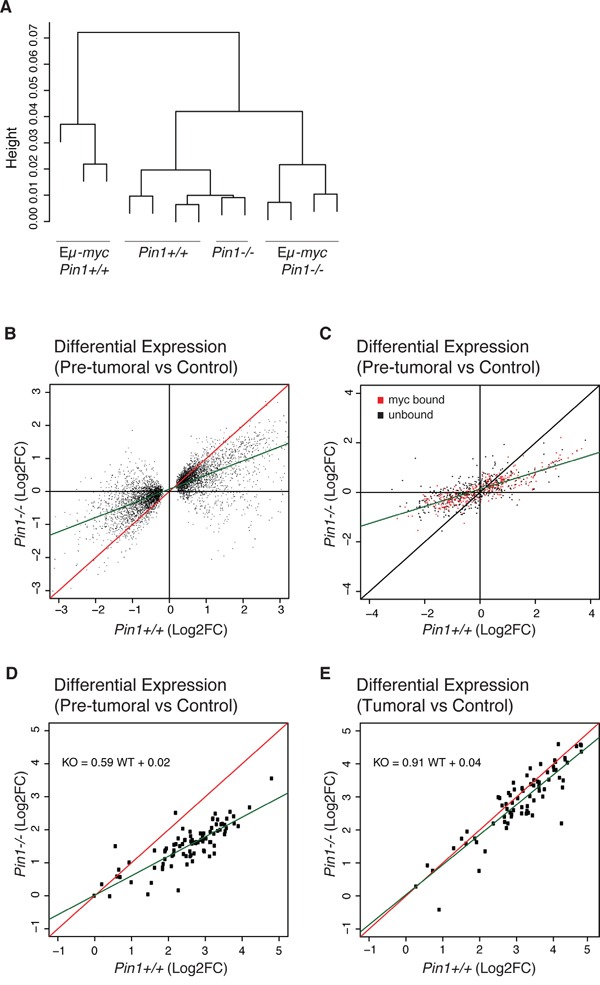
Gene-expression profiling in Eμ-*myc Pin1−/−* B cells Total RNA from control and pre-tumoral Eμ-*myc* B cells of the indicated *Pin1* genotypes was profiled by RNA-seq. **A.** Unsupervised hierarchical clustering of the sequenced samples. **B.** Fold-change values (log2FC) for differentially expressed genes (DEGs) in the *Pin1^−/−^* relative to the *Pin1^+/+^* background. The DEGs shown here were first defined based on their deregulation in Eμ-*myc* B relative to control B cells in the *Pin1*+/+ background (see Methods). **C.** 754 genes covering the whole expression range and regulatory patterns in Eμ-*myc* B cells [40] were analyzed by NanoString and reported as in B. The data are based on the average of 3 biological replicates for each genotype. 361 genes previously classified as Myc-bound in pre-tumoral B cells [40] are represented in red, and 393 unbound genes in black. **D., E.** NanoString analysis of 80 genes that are amongst the most strongly induced in pre-tumoral B cells and are all bound by Myc in their promoter regions [40]: D. and E. show the fold-changes in pre-tumoral B cells and lymphomas, respectively, both relative to control B cells. The green lines in B., C., D. represent the linear regression of the data.

Pin1 may also modulate Myc's transcriptional activity [17, 18]. To address this issue, we generated RNA-seq profiles from control and pre-tumoral B cells of either *Pin1* genotype. Unsupervised hierarchical clustering led to the grouping of all non-transgenic samples with no distinction of *Pin1* genotype, implying that Pin1 does not regulate basal transcription in B cells (Figure 4A). Eμ-*myc Pin1^+/+^* and Eμ-*myc Pin1^−/−^* samples formed distinct groups, the latter clustering closer to non-transgenic cells. Consistent with this scenario, gene activation and repression detected in Eμ-*myc Pin1^+/+^* pre-tumoral relative to *Pin1^+/+^* control B cells [40] were generally reduced in the *Pin1^−/−^* background (Figure 4B). Digital quantification of 754 mRNAs with NanoString technology confirmed this general dampening in their up- or down-regulation, independently from Myc binding to the corresponding promoters [40] (Figure 4C). We further examined a set of 80 genes that were Myc-bound and strongly induced in Eμ*-myc* B cells [40]: as above, induction of these genes was reduced - albeit still detectable - in pre-tumoral *Pin1^−/−^* B cells, but was fully restored in *Pin1^−/−^* lymphomas (Figure 4D, 4E), indicating that Pin1 was not required for Myc transcriptional activity *per se*. This issue was addressed further in MEFs, with a set of 64 MycER-induced or -repressed mRNAs [40] revealing comparable responses in *Pin1^−/−^* and *Pin1^+/+^* MEFs (Supplementary Figure 3A). Moreover, a group of 116 Myc-Dependent Serum Response (MDSR) genes [29] also showed unaltered responses to serum stimulation in the *Pin1^−/−^* background (Supplementary Figure 3B). Consistent with this result, loss of Pin1 did not suppress binding of Myc to the *Ncl* promoter shortly after serum stimulation (Supplementary Figure 3C) [29, 41]. Altogether, our data show that Pin1 exerts no essential role in Myc-dependent transcription. We infer that the impaired gene regulation seen in pre-tumoral Eμ-*myc Pin1^−/−^* B cells - including reduced expression of the Eμ-*myc* transgene itself - most likely follows from their quiescent state. In line with this interpretation, two cyclin-coding (*Ccnb1* and *Ccnd1*) and 28 other cell cycle-associated genes showed lower expression in Eμ-*myc Pin1^−/−^* relative to Eμ-*myc Pin1^+/+^*B cells (Supplementary Figure 3D, 3E). In Eμ-*myc Pin1^−/−^* lymphomas, instead, expression of these genes was back to the levels observed in the *Pin1^+/+^* counterparts (Supplementary Figure 3E).

**Figure 5:**
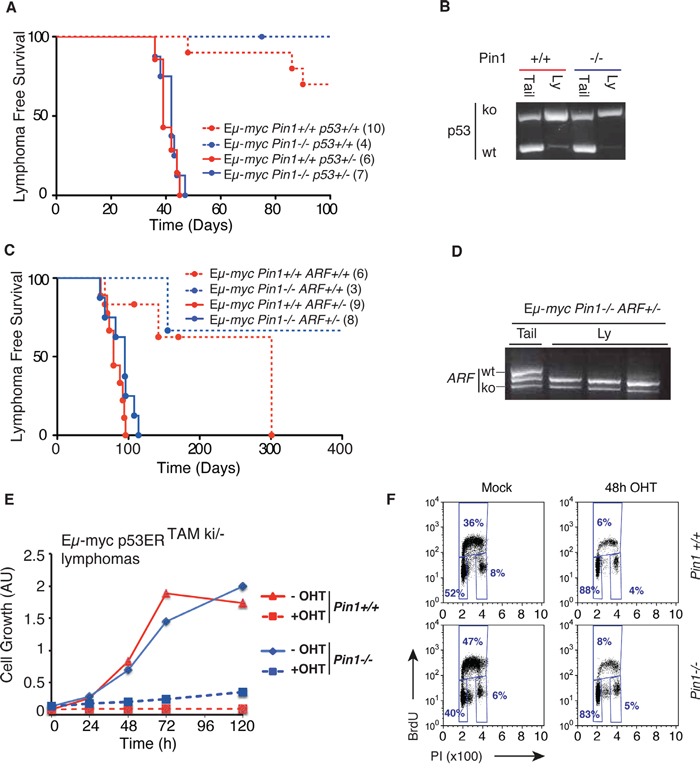
Delayed lymphomagenesis in the *Pin1−/−* background requires Arf/p53 activity **A.** Lymphoma-free survival in cohorts of Eμ-*myc* or control mice of the indicated Pin1 and p53 genotypes. The median survival was 39 days for Eμ-*myc Pin1^+/+^*p53*^+/−^* and 42 days for Eμ-*myc Pin1^−/−^*p53*^+/−^* mice (P=0.5959, Log-Rank, Mantel-Cox). Numbers within brackets indicate sizes of each cohort. **B.** Lymphomas arising in Eμ-*mycp53^+/−^* mice show p53 loss of heterozygosis (LOH) in either *Pin1^+/+^* or *Pin1^−/−^* background, as shown by RT-PCR. One example of each is shown. Three Eμ-*myc p53^+/−^*Pin1*^−/−^* tumors were analyzed with the same outcome. **C.** Same as A. with the indicated *Pin1* and *ARF* genotypes. Calculated Median survival was 79 days for Eμ-*myc Pin1^+/+^*ARF*^+/−^* and 95 days for Eμ-*myc Pin1^−/−^*ARF*^+/−^* mice (P=0.0959, Log-Rank, Mantel-Cox). Note that in both A. and C., Eμ-*myc Pin1^−/−^* mice with functional p53 and Arf show delayed lymphomagenesis relative to their *Pin1^+/+^* counterparts, validating the results shown in Figure 1A. **D.** Lymphomas arising *in* Eμ-*mycPin1^−/−^ARF^+/−^* mice show ARF loss of heterozygosis (LOH). Three tumors and one tail from Eμ-*myc Pin1^−/−^*ARF*^+/−^* mice were analyzed by RT-PCR. **E.** Growth curves of Eμ-*myc* p53ER^TAMki/−^ lymphomas overexpressing the anti-apoptotic protein Bcl2. Cells were cultured either in the presence (100 nM) or absence (Mock) of OHT. Cell growth was assessed with the Cell Titer Glo assay. **F.** Cell cycle analysis of Bcl2-expressing Eμ-*myc* p53ER^TAMki/−^ lymphomas 48 hours after OHT administration.

The ARF/p53 pathway is an important sensor of Myc activation, and is required for tumor suppression in Myc-induced lymphomas [16, 27]. Stabilization and activation of p53 by genotoxic stress involves phosphorylation of Ser-Pro motifs and enhanced interaction with Pin1, and *Pin1 null* MEFs are defective in DNA Damage-induced G2 arrest [11, 12]. Based on these observations, one might have expected impaired p53 activity and accelerated lymphomagenesis in Eμ-*myc Pin1^−/−^* mice, instead of the observed delay (Figure 1A). To address whether this delay was ARF- or p53-dependent, we bred Eμ-*myc Pin1^−/−^* mice into either of the p53*^+/−^* or *ARF^+/−^* backgrounds, both known to dramatically accelerate Myc-induced lymphomagenesis with loss of the remaining p53 or ARF allele [16, 42]. Most importantly, these effects were still observed in the *Pin1^−/−^* background, while the delay in lymphomagenesis relative to *Pin1^+/+^* counterparts was lost (Figure 5A-5D). Inactivation of the ARF-p53 pathway may occur spontaneously during lymphomagenesis in Eμ-*myc* mice and, as assayed by immunoblot analysis, also occurred in Eμ-*myc Pin1^−/−^* mice (Supplementary Figure 4A). Hence, the selective pressure against the ARF/p53 pathway was intact in the absence of Pin1 and, once having lost p53 activity, lymphomas were no longer delayed by the lack of Pin1. In an analogous manner, following knockdown of p53, MycER activation no longer slowed proliferation of *Pin1^−/−^* MEFs (Supplementary Figure 4B). Altogether, our data show that, Pin1 is required to prevent the build-up of an ARF/p53-dependent cytostatic response upon oncogenic activation of Myc.

To further assess how Pin1 influences p53 activity in lymphomas, we used tumors derived from Eμ-*myc* mice expressing p53ER^TAM^, a latent, OHT-inducible form of p53: as expected [43], OHT treatment induced an acute apoptotic response in those tumors, which was not affected by loss of Pin1 (Supplementary Figure 4C). Over-expression of the anti-apoptotic protein Bcl2 caused a switch in the response to p53ER^TAM^ activation, with cells undergoing immediate cell cycle arrest: most importantly, this was also independent from the *Pin1^−/−^* background (Figure 5E, 5F). Hence, Pin1 had no direct impact on either the apoptotic, or the growth inhibitory activity of p53 in these B cell lymphomas.

**Figure 6:**
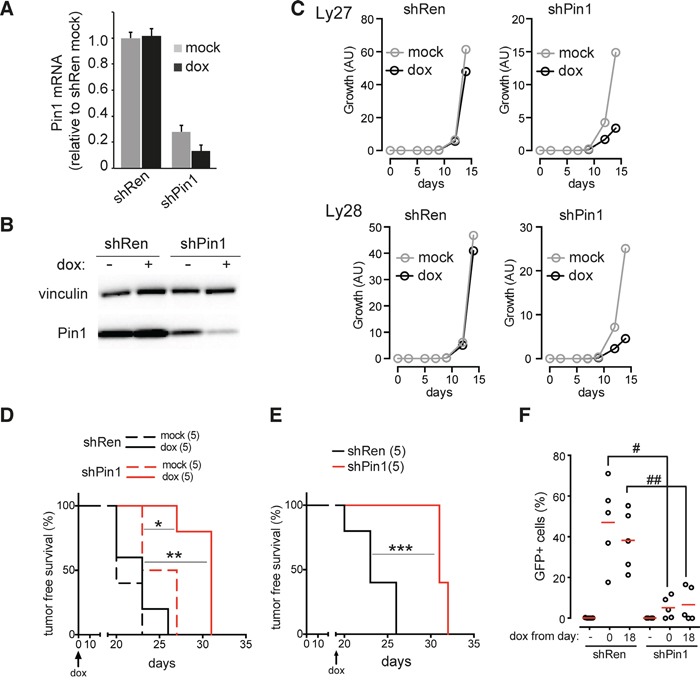
Pin1 silencing impairs tumor growth and dissemination **A.** RT-qPCR of Pin1 mRNA. **B.** Immunoblot analysis of Pin1 protein levels, respectively, in a primary Eμ-*myc* lymphoma infected with conditional shRNAs targeting either Pin1 (shPin1) or renilla luciferase (shRen). The shRNAs were induced by supplementing cells with 1 μg/mL doxycycline for 24 hours. The mRNA data represent the averages ±s.d. of three technical replicates, all normalized to the housekeeper TBP and to the mock-treated shRen lymphoma. Vinculin was used as a loading control. **C.** Growth of two independent primary lymphoma populations (Ly27 and Ly28) infected with either shRen or shPin1, and cultured with (dox) or without (mock) doxycycline. **D., E.** Tumor-free survival in mice transplanted with an shRen or shPin1-bearing lymphoma (Ly28). Doxycycline was administered either continuously from the time of transplantation (D.) or following detection of tumor masses, 18 days after transplantation (E.). **F.** Dot plot showing the residual percentage of GFP-positive B220^+^ tumor cells detected in the tumor-infiltrated lymph nodes of animals transplanted with shPin1 or shRen lymphomas. Red bars indicate the average values. As in D., E., Mice were fed with doxycycline starting from days 0 or 18, as indicated. GFP serves as a marker for the doxycycline-dependent induction of the shRNAs. * p=0.014; ** p=0.0022; *** p=0.0026 (Log-rank, mantle-cox); **^#^** p=0.002, **^##^** p=0.003 (t-test).

We next asked whether Pin1 would be required for the growth of established lymphomas. To this end, we used an shRNA (shPin1) inserted in a retroviral vector allowing to conditionally silence Pin1 in a doxycycline-dependent manner (Supplementary Figure 5A). Despite a basal leakiness of the shPin1 construct, causing a reduction in Pin1 levels relative to a control shRNA (shRen), administration of doxycycline further reduced Pin1 expression (Figure 6A, 6B), reducing cell proliferation *in vitro* in two independent Eμ-*myc* lymphomas (Figure 6C). This was not due to toxicity of doxycycline at the concentrations used, as the antibiotic had no significant effect on shRen-infected cells. To address the role of Pin1 in tumor dissemination *in-vivo*, recipient mice were orthotopically transplanted with an Eμ-*myc* lymphoma infected with either shRen or shPin1. While a doxycycline diet did not have any effect on the expansion of *in-vivo* growth of the lymphomas infected with shRen, the expansion of shPin1-infected tumors was markedly delayed, significantly improving survival of the animals (Figure 6D). A similar effect occurred upon silencing of Pin1 in already established lymphomas (Figure 6E). In both instances, those shPin1 lymphomas that ultimately arose in doxycycline-fed animals showed a remarkably low percentage of GFP positive cells (Figure 6F): as GFP and the shRNA are expressed from the same precursor RNA (Supplementary Figure 5A), this result demonstrates selective outgrowth of lymphoma cells that escaped Pin1 silencing. Thus, down-regulation of Pin1 in tumors leads to a strong impairment in cancer cell dissemination and maintenance.

## DISCUSSION

To date, the genetic interaction between Myc and Pin1 was addressed only in a mouse model of mammary carcinogenesis, in which Pin1 was dispensable for Myc-induced tumorigenesis [44]. Here, we have shown that Pin1 has a critical role in Myc-driven B cell lymphomagenesis in the Eμ-*myc* transgenic model, in line with its tumor-promoting activity in other cell types and in the context of Ras signaling [45, 46]. This effect of Pin1 was not due to its known effects on Myc and p53 activities [11, 12, 39], which were not directly altered in the *Pin1*^−/−^ background.

Based on previous evidence, the absence of Pin1 should have caused defective de-phosphorylation of Myc Serine 62, a consequent increase in the Ser 62/Thr 58/ phosphorylation ratio, and stabilization of the protein [7, 39]. Surprisingly, however, neither B-cells nor MEFs showed clear evidence for an alteration of these features in the *Pin1 ^−/−^* background. Two experimental features are noteworthy here: first, the experiments that established the Ser 62/Thr 58/ phosphorylation cycle were based on Myc overexpression with an Adenoviral vector in serum-stimulated fibroblasts [7], but no data conclusively addressed the role of Pin1 in phosphorylation of the endogenous Myc protein, as done here in *Pin1^−/−^* fibroblasts and B cells. Second no data were available concerning the identity of the Ub-ligase responsible for Myc degradation following Ser 62 de-phosphorylation [7]: based on independent work [20, 21], this is commonly surmised to be the T58P-dependent ligase Fbw7 [39, 47]. However, the original data on Fbw7 indicated that this enzyme may associate with Thr58-phosphorylated Myc independently from Ser 62 dephosphorylation [20, 21]. It remains to be addressed whether redundant effects exist that compensate for the loss of Pin1 in our experiments. Other data indicated that Pin1 might be critical for the transcriptional activity of Myc [17]: once again, however, our analysis failed to unravel any defect in Myc-dependent transcription in the *Pin1^−/−^* background, in either B cells or MEFs. We are thus facing a complex regulatory circuitry, in which deletion of a component - here Pin1 - may not have obvious consequences.

In line with the preserved transcriptional activity of Myc in *Pin1^−/−^* cells, Pin1 was required for neither the mitogenic, nor the pro-apoptotic activities of Myc. Upon oncogenic activation of Myc, however, Pin1 was required to prevent the build-up of an ARF/p53-dependent cytostatic response, observable both in *Pin1^−/−^* MEFs and Eμ-*myc Pin1^−/−^* mice. Albeit strongly reminiscent of oncogene-induced senescence [36], which can become limiting for Myc-induced lymphomagenesis in certain genetic backgrounds [30, 48, 49], this effect lacked any of the canonical features associated with senescent cells. The direct targets of either Myc or Pin1 required to exert this effect remain to be characterized, albeit both regulate genes and proteins involved in a diversity of fundamental biological processes, such as transcription, mRNA stability, cell division, growth, differentiation, stress responses, aging and survival [4, 50], many of which may participate to the effects reported here.

Finally, in line with our data in *Pin1^−/−^* mice, knockdown of Pin1 in Eμ-*myc* lymphomas impaired their proliferations *in vitro* and significantly enhanced survival of disease-bearing mice. A most interesting implication from these data is that pharmacological inhibition of Pin1 might lead to the proliferative arrest - and possibly elimination - of Myc-driven lymphomas: initial experiments with the Pin1 inhibitory compound PiB [51] were inconclusive, as this molecule showed off-target effects, inhibiting proliferation of Pin1-knockout as well as wild-type Eμ-*myc* tumors (data not shown). Our findings provide an ideal pre-clinical setting in which to characterize the activity and specificity of new Pin1-inhibitory compounds.

## MATERIALS AND METHODS

### Mouse breeding, genotyping, and analysis

Eμ-*myc* transgenic mice [24] and *Pin1*^+/−^ [23] mice were bred to obtain the various genotypic combinations described in this paper. Mice were maintained on a C57BL/6 background and genotyped as described [23, 35]. Cohorts of Eμ-*myc* mice with *Pin1^+/+^*, *Pin1*^+/−^ or *Pin1*^−/−^ backgrounds were monitored twice a week for lymphoma development by peripheral lymph-node palpation. For pre-tumoral analysis, 6–8 weeks old mice with no infiltration of peripheral lymph-nodes were used. Percentages and numbers of blood cell populations were measured using a Hematological analyzer (Beckman Coulter). For Tunel assay, purified B cells were fixed for 10 min in 2% formaldehyde, permeabilized in 0.1% triton in PBS, and analyzed with the ApopTag Plus fluorescein in situ Apoptosis detection kit (Millipore). Immuno-histochemical analysis was performed as described [30] with Monoclonal Rat Anti-Mouse Ki-67 Antigen Clone TEC-3 (DakoCytomation), and Polyclonal Rabbit Anti-Rat Immunoglobulins/HRP. The EnVision+ system (DakoCytomation) was used for signal detection. Immunoblot analysis was performed as described [30] with the following antibodies: rabbit polyclonal Myc N100 (custom-made antibody raised against the first 100 N-terminal aminoacids of c-Myc); anti c-Myc Y69 (abcam 32072); anti Ser 62-phosphorylated Myc (S62P-Myc) [52]; anti Thr 58-phosphorylated Myc (T58P-Myc) (Santa Cruz-135647); rabbit polyclonal anti-Pin1 [11]; anti-phospho-Histone H2A.X (Ser 139, 1:2000, Millipore); anti-p53 (1C12, 1:1000 Cell Signaling), anti-p53 (Ser15, 1:1000, Cell Signaling); anti-vinculin (Sigma). For preclinical studies, 10^6^ cells derived from Eμ-*Myc* lymphomas were transplanted into syngeneic C57/Bl6 mice by tail vein injection. In order to induce the knockdown, recipient mice were fed with doxycycline-containing food either at the day of injection or when tumors became palpable. A control group of mice and some of the tumor-bearing mice were kept on standard doxycycline free diet. Mice were inspected 3 times a week for the insurgence of tumor masses.

### Construction of the conditional Pin1 shRNA vector

To generate shRNA targeting Pin1 (ShPin1), we cloned a 97-mer oligonucleotide (TGCTGTTGACAGTGAGCGCCACAGTATTTATTGTTCCTAATAGTGAAGC CACAGATGTATTAGGAACAATAAATACTGTGTTGC CTACTGCCTCGGA) designed by Dr. Johannes Zuber based on the massively parallel sensor based assay [53]. The 97mer served as a template for a PCR reaction performed using the using the primers miRE-Xho-fw (TGAACTCGAGAAGGTATATTGCTGTTGACAGTGAGCG) and miRE-EcoOligo-rev (TCTCGAATTCTAGCCCCTTGAAGTCCGAGGCAGTAGGC).

Primary Eμ-*myc* lymphomas were infected *in vitro* with viral supernatant containing either shPin1 or shRen and grown *in vitro* in tet-free medium. Cell growth was monitored using the CellTiter-Glo Luminescent Cell Viability Assay (Promega). The shRNAs were induced *in vitro* by supplementing cells with 1 μg/mL doxycycline or *in vivo* with doxycycline-containing food.

### Flow cytometry and magnetic cell sorting of B-lymphocytes

For the analysis of B cell populations in pre-tumoral mice, bone marrow, splenic or white blood cells were incubated for 30 min with anti-CD45R/B220 PE, CD25-APC, B220-Cy7PE, c-kit-PE (BD Biosciences Pharmingen) and with fluorescein isothiocyanate (FITC)-conjugated anti-IgM (Jackson Immunoresearch). For cell cycle analysis, after surface staining, cells were fixed 10 min in 2% formaldehyde at RT, permeabilized with Cytofix/Cytoperm Kit (BD Biosciences Pharmingen) and incubated for 45 min with anti-Ki67/Alexa488 (BD Biosciences Pharmingen). Cells were fixed before of Hoechst staining. To measure ROS, cells were incubated with CellROX deep Red (Molecular Probes, C10422) and analyzed as described by the manufacturer. FlowFISH was performed using the Telomere PNA Kit/FITC for Flow Cytometry DAKO, as described by the manufacturer. Sample and control cells were washed in PBS, denaturated for 10 min at 80°C. Hybridization with Telomere PNA Probe/FITC was performed overnight. After two washing steps, cells were stained with PI in the dark at 2-8°C for 3 hours before of FACS acquisition. After flow cytometric analysis, the data obtained were used for the determination of the relative telomere length (RTL), calculated as the ratio between the telomere signal of each sample and that in the control 1301 cell line, with correction for the G0/G1 DNA index, as described by the manufacturer.

Splenic B cells for RNA and protein preparation were isolated as described [35]. For cell culture, we used the MACS B cell Isolation kit (Miltenyi Biotech). Purified B cells were plated in B cell medium (DMEM and IMDM in ratio 1:1, 10% fetal bovine serum, 1% glutamine, 1% penicillin/streptomycin and 25uM Δ-mercaptoethanol) supplemented with 20 μg/ml LPS (Sigma L6529). Primary lymphomas were cultured in B cell medium, previously conditioned by irradiated (30Gy) NIH-3T3 cells. BrdU incorporation was analyzed as described [30]. MEFs derived from E12.5 embryos were infected with retroviruses encoding the MycER chimaera [54] and analyzed as previously described [30]. Cell death in MEFs cultures was assessed with the Caspase-Glo 3/7 kit (Promega), while cell growth was monitored using the CellTiter-Glo Luminescent Cell Viability Assay (Promega).

### RNA extraction and analysis

Total RNA purified using the Quiagen RNeasy Mini Kit was processed for RNA-seq, and the data analyzed as described [40]. The data are reported in Supplementary Table 1. Genes with a maximum expression value of RPKM ≥ 2 were hierarchically clustered with the R function “hclust” (Figure 4A). Differentially expressed genes (DEGs) were defined in the *Pin1^+/+^* background as genes up- or down- regulated in pre-tumoral Eμ-*myc* relative to control B cells, with q-value < 0.05 and a maximum expression value of RPKM ≥ 2. For the identified DEGs the gene expression changes in pre-tumoral relative to control B cells (Figure 4B) was calculated as the ratio between the mean of RPKM values in Pre-tumoral B cells and the mean of RPKM values in control B cells. The log2 of the calculated values in Pin1+/+ (X-axes) and in Pin1−/− (Y-axes), were reported in the scatter-plot (Figure 4B). 100 ng of total RNA were processed for NanoString analysis as described by the manufacturer. The custom-made CodeSets used here as reported in Supplementary Tables 2-5, and the data in Supplementary Tables 6-9. Two of the CodeSets (Supplementary Tables 2, 4) were the same as those used in our previous work [40]. Dedicated nCounter software was used for data analysis, and raw counts were normalized to the internal positive control probes, included in each CodeSet, and to the housekeeping gene TBP. RNAseq data have been deposited and are accessible through GEO Series accession number GSE77482 (https://www.ncbi.nlm.nih.gov/geo/query/acc.cgi?acc=GSE77482).

## SUPPLEMENTARY FIGURES AND TABLES




