# Supplementary material for: Pin1 is required for sustained B cell proliferation upon oncogenic activation of Myc
Source: Oncotarget. 2016 Mar 2;7(16):21786–98. doi: 10.18632/oncotarget.7846 (PMC5008323; doi:10.18632/oncotarget.7846)
Supplement: Supplementary file 1 [file oncotarget-07-21786-s001.pdf]

SUPPLEMENTARY FIGURES AND TABLES

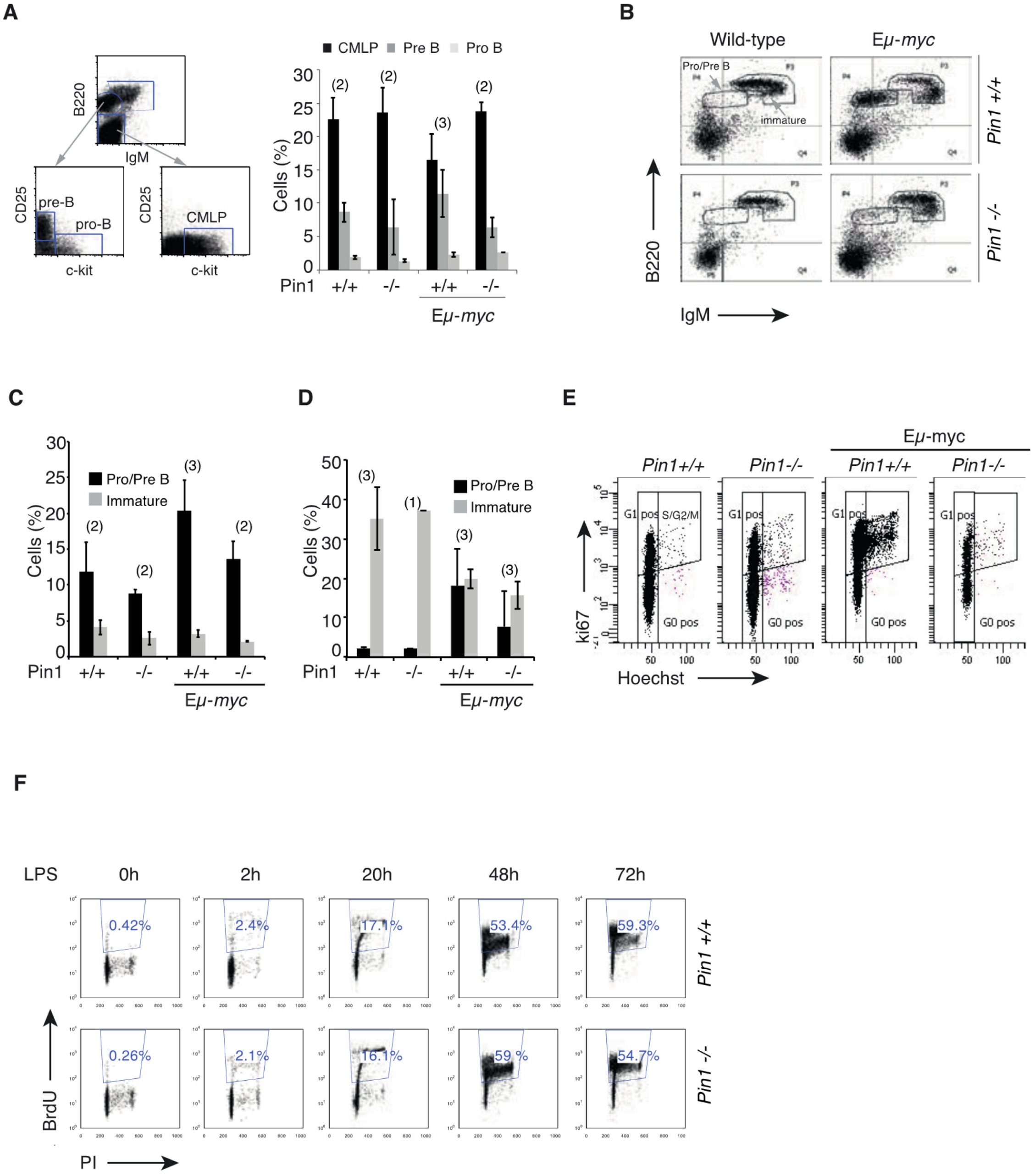

Supplementary Figure S1: Analysis of control and pre-tumoral *Pin1*$^{-/-}$ B cells. A. Flow-cytometric (FACS) analysis of bone marrow B cells isolated from mice of the indicated genotypes. The FACS profiles on the left as provided as an illustrative example: Common myeloid/lymphoid precursors (B220$^-$IgM$^-$CD25$^-$c-kit$^+$) Pro B (B220$^+$IgM$^-$CD25$^-$c-kit$^+$) and Pre B (B220$^+$IgM$^-$CD25$^+$c-kit$^-$) cells [55] were identified as indicated. **B.** FACS analysis of peripheral blood in control non-transgenic and pre-tumoral Eμ-*myc* mice.Pro/Pre B lymphocytes are defined as B220$^+$IgM$^-$ and Immature B lymphocytes as B220$^+$IgM$^+$ cells. Representative profiles are shown here, and the quantification of multiple measurements in Figure 1B. **C, D.** Summary of FACS analyses of bone marrow (C.) and splenic B cells (D.), analyzed as in B. **E.** Cell cycle analysis of circulating B cells. Cells were stained with antibodies against B220 and then processed for cell cycle analysis using antibodies against the proliferation marker Ki67 and the DNA-content dye Hoechst. Representative Ki67/Hoechst FACS profiles of B220$^+$-gated cells are shown, allowing us to distinguish cells in G0 (Ki67$^-$), G1 (Ki67$^+$/1N DNA content) and S/G2/M (Ki67$^+$/2N DNA content). B cells in non-transgenic mice were largely quiescent, regardless of the Pin1 genotype. **F.** Proliferation of purified B cells cultured *in vitro* in the presence of LPS, as assessed by continuous labeling with BrdU. At the reported time-points, the percentage of BrdU-positive cells was determined by FACS, as shown. The number of animals analysed are indicate within brackets.

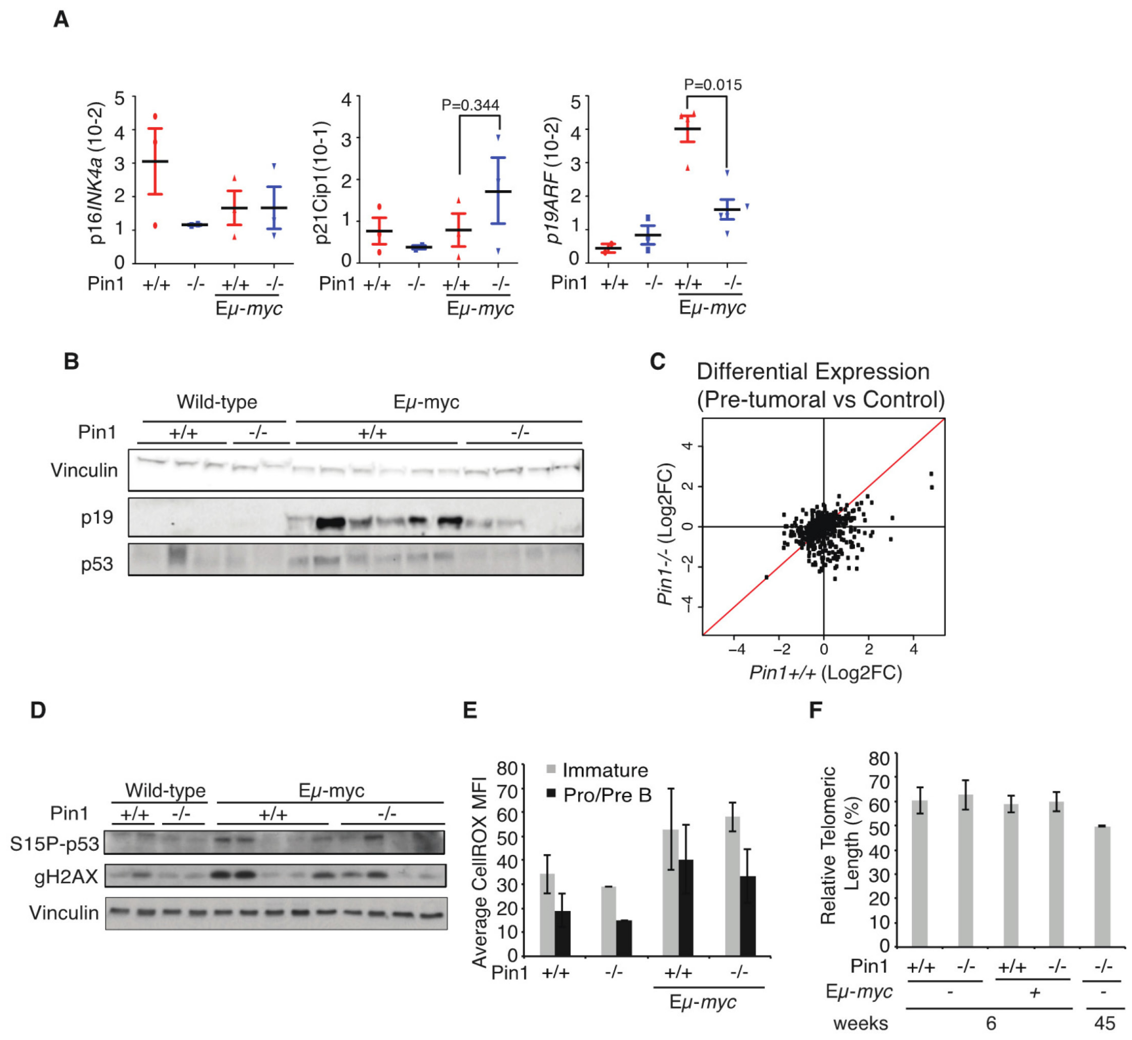

Supplementary Figure S2: Analysis of senescence markers in pre-tumoral Eμ-*myc Pin1*$^{-/-}$ mice. A. RNA from control and pre-tumoral splenic B cells of the indicated Pin1 genotypes were analyzed by quantitative RT-PCR for the indicated mRNAs, all normalized to TBP. The indicated P-values were calculated using Student's t-test. **B.** Immunoblot analysis of p19^{ARF} and p53 in splenic B220^{+} cells in mice of the indicated genotypes. Vinculin is used as loading control. **C.** Fold-change values (log2FC) for p53 target genes [56] in the *Pin1*$^{-/-}$ relative to the *Pin1*$^{+/+}$ background. The data for this set of genes were extracted from our RNAseq dataset. **D.** Immunoblot analysis of the DNA Damage Response markers pSer15-p53 and γH2AX in splenic B cells of control and pre-tumoral mice. **E.** Mean fluorescence intensity of the CellROX Deep Red probe was measured in B cells by flow cytometry. The average value of three mice is reported. **F.** Relative Telomeric length in splenic B220^{+} cells of the indicated genotypes, as assayed by Flow Fish [57]. Data represent the average ±s.d. of three samples per genotype. A 45 week-old *Pin1*$^{-/-}$ mouse was used as control.

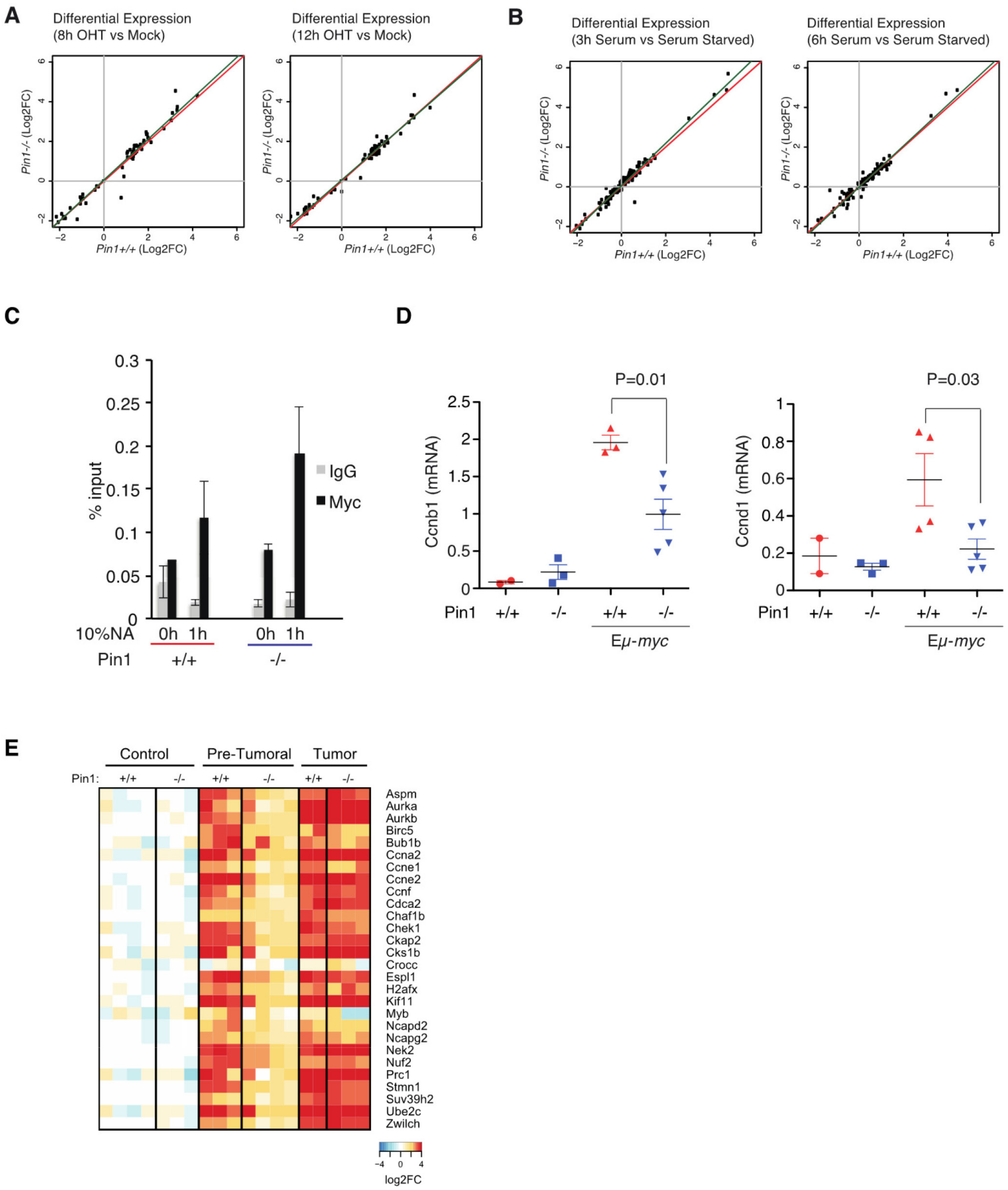

Supplementary Figure S3: Myc dependent transcription and in MEFs and B cells. A. NanoString-based mRNA quantifications for 64 MycER-responsive genes [40]. **B.** NanoString-based mRNA quantifications for 116 Myc-Dependent Serum Response genes [29]. Fold-changes (Log2FC) for each mRNA are reported in *Pin1*$^{-/-}$ relative to *Pin1*$^{+/+}$ MEFs. The data represent the averages from two independent measurements for each genotype. **C.** Myc binding to the *Ncl* promoter upon serum stimulation in either *Pin1*$^{+/+}$ or *Pin1*$^{-/-}$ MEFs was measured by ChIP-qPCR. Data represent the average ±s.d. deviation of triplicate measurements. **D.** *Ccnb1* and *Ccnd1* mRNAs assessed by quantitative RT-PCR and normalized to TBP mRNA in control and pre-tumoral splenic B cells of the indicated Pin1 genotypes. P-values were calculated using Student's t-test. **E.** Analysis of cell cycle-related mRNAs with NanoString technology. The heatmap shows the fold-change (log2FC) between the NanoString count in each sample and the mean of the counts in control Pin1$^{+/+}$ B cells. The genes included here are those belonging to the cell cycle category (GO:0007049) within the larger set analyzed in Figure 4D., 4E.

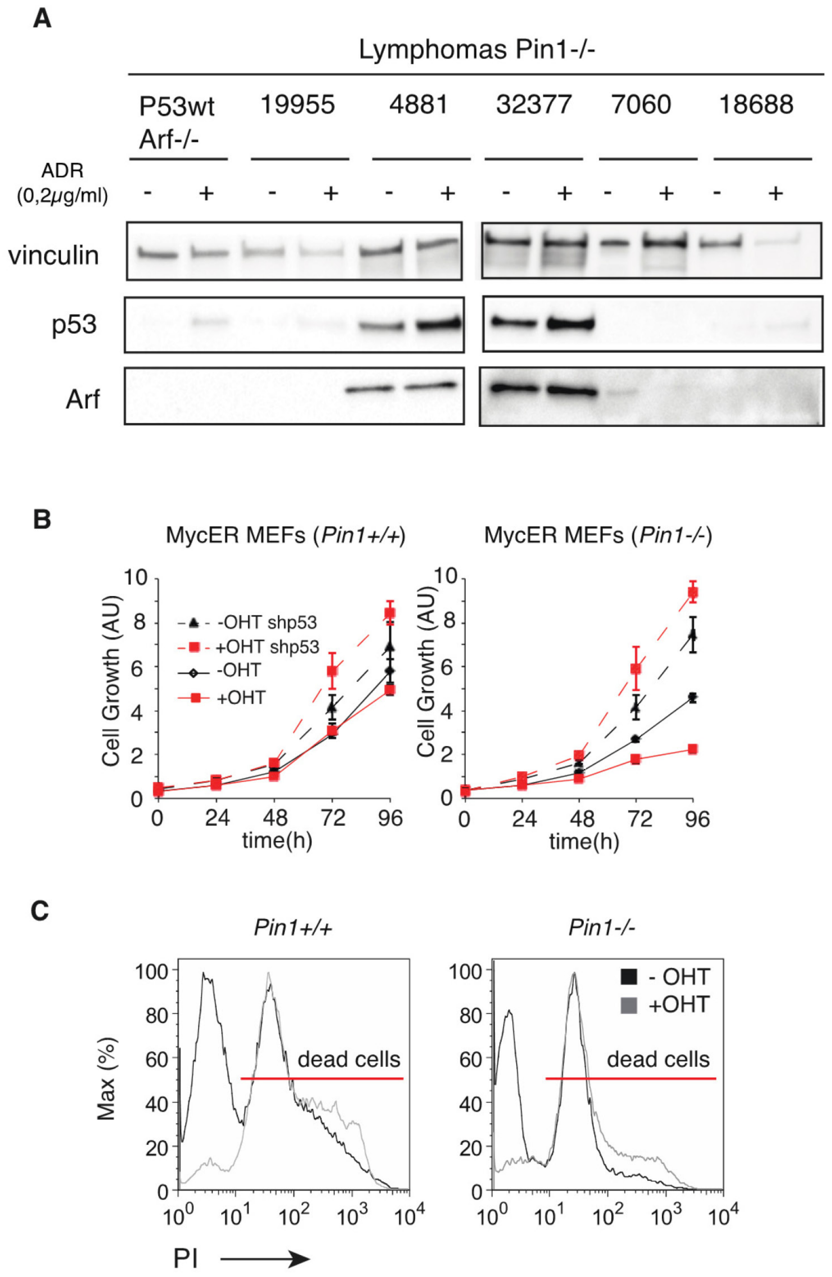

Supplementary Figure S4: p53 activity in *Pin1*$^{-/-}$ cells. A. Immunoblot analysis of Arf and p53 in cell lysates prepared from cultured lymphomas exposed to Adriamycin (ADR, 0,2 μg/ml for 5 hours) or left untreated. The first two lanes show a lymphoma derived from an Eμ-*myc ARF*$^{+/-}$ mouse that spontaneously inactivated the wild-type ARF allele, preserving wild-type p53 activity: p53 is expressed at physiological levels and is responsive to Adriamycin in these cells. Five independent *Pin1*$^{-/-}$ lymphomas were analyzed. Lymphomas 4881 and 32377 accumulated p53 to supra-physiological levels in the absence of exogenous DNA damaging agents, indicative of p53 mutations. Consistent with loss of the negative feedback on Arf expression, these tumors also accumulated Arf to elevated levels [16]. Lymphomas 19955 and 18688 showed physiological levels of p53 and no detectable Arf, most likely owing to spontaneous loss of Arf. Lymphoma 7060 showed expression of neither p53, nor Arf. **B.** *Pin1*$^{+/+}$ and *Pin1*$^{-/-}$ MycER-MEFs were infected with retroviruses expressing shRNA against murine p53 (shp53). Cells were treated with OHT or ethanol, and cumulative cell growth monitored for 96 hours. The data represent the average ±s.d. of a triple measurement, expressed as arbitrary units (AU). Consistent results were obtained with two biological replicates for each Pin1 genotype. **C.** Eμ-*myc* p53ER$^{TAMki/-}$ lymphomas were cultured in the presence of 100 nM OHT. After 8 hours, dead cells were quantified based on their permeability to Propidium Iodide (PI). PI incorporation was measured by flow cytometric analysis.

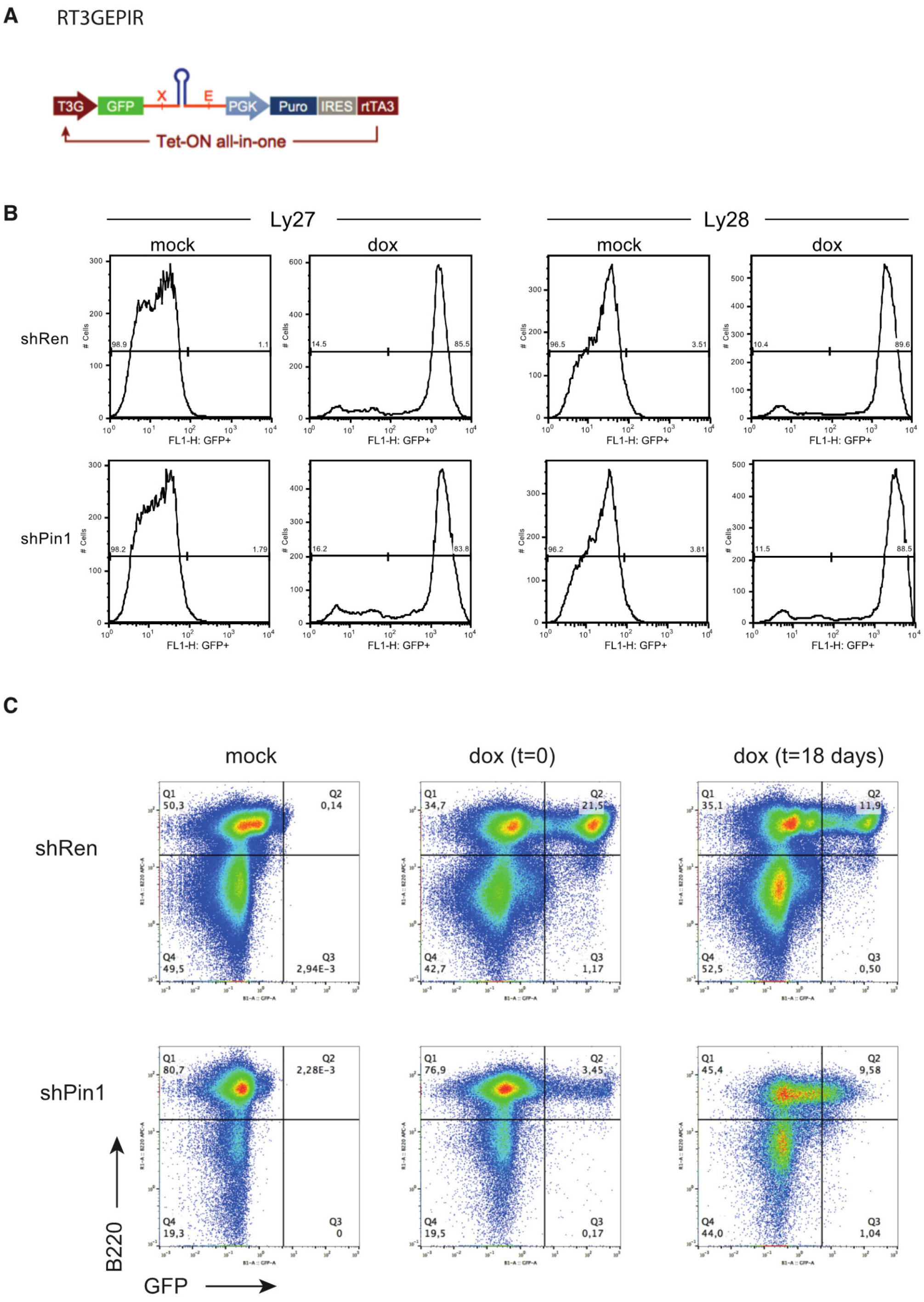

Supplementary Figure S5: Schematic view of the shRNA vector. A. Conditional vector used for silencing Pin1, based on the tet-inducible vector RT3GEPIR [58]. DNA encoding for the short RNA hairpin is represented in blue. **B.** FACS analysis of GFP expression, used as marker for the activation of the shRNAs, in Eμ-myc lymphomas infected with either shPin1 or shRen. Cells were grown *in vitro* for 48 hours with either doxycycline (dox) or tet-free medium (mock). **C.** FACS profiles of representative shRen and shPin1 lymphomas derived from animals fed with either regular diet (mock) or food supplemented with doxycycline (dox).

Supplementary Table S1: RNA-seq profiling in Eμ-myc mice

See Supplementary File 1

Supplementary Table S2: NanoString CodeSet design for the Eμ-myc model

See Supplementary File 1

Supplementary Table S3: NanoString CodeSet design for Top Myc bound and induced genes the Eμ-myc model

See Supplementary File 1

Supplementary Table S4: NanoString CodeSet design for the Myc-ER model

See Supplementary File 1

Supplementary Table S5: NanoString CodeSet design for the Myc Dependent Serum Response (MDSR) model

See Supplementary File 1

Supplementary Table S6: NanoString profiling in Eμ-myc

See Supplementary File 1

Supplementary Table S7: NanoString profiling of top Myc bound and induced genes in the Eμ-myc model

See Supplementary File 1

Supplementary Table S8: NanoString profiling in MycER MEFs

See Supplementary File 1

Supplementary Table S9: NanoString profiling in the MDSR model

See Supplementary File 1
